# Innovative Techniques and Outcomes in Pediatric and Neonatal Thoracic Surgery: A Comprehensive Narrative Review of Current Practices and Future Directions

**DOI:** 10.7759/cureus.91294

**Published:** 2025-08-30

**Authors:** Ahmed Alemam, Mohamed Abosheisha, Rezuana Tamanna, Momen Abdelglil, Mohamed Ali, Ahmed Swealem, Samir Bin Halim, Md Abdus Samad Hasan, Mohamed Ismaiel

**Affiliations:** 1 General Surgery, Leicester Royal Infirmary, University Hospitals of Leicester, Leicester, GBR; 2 General Surgery, Watford General Hospital, Watford, GBR; 3 Pediatric Surgery, Mansoura University Children Hospital, Mansoura, EGY; 4 Surgery, Hywel Dda University Health Board, Carmarthen, GBR; 5 Orthopedics, North Bristol NHS Trust, Bristol, GBR; 6 General Surgery, Bangkok Hospital, Bangkok, THA; 7 General Surgery, University Hospital Limerick, Limerick, IRL

**Keywords:** congenital diseases, lung malformations, minimally invasive, pediatric surgical advancements, pediatric thoracic surgery, thoracoscopic surgery

## Abstract

Pediatric thoracic surgery has undergone significant changes and improvements due to the evolution of minimally invasive techniques, robotic-assisted interventions, and enhanced patient care. Enhanced thoracic surgical interventions progressed in pediatric thoracic tumors, airway management, chest wall reconstruction, and lung transplantation. Key areas for improvement include the adoption of enhanced recovery protocols, enhancing long-term outcomes, and integrating emerging technologies such as 3D printing and artificial intelligence. Despite the advancement in these fields, challenges still exist, underscoring the importance of high and specialized training, multidisciplinary collaboration, and future and continued research to optimize patient outcomes and shape the future of pediatric thoracic surgery. Emerging evidence supports standardized perioperative pathways tailored to children, including multimodal opioid-sparing analgesia, early mobilization, and proactive pulmonary physiotherapy. Advances in imaging and intraoperative navigation are refining lesion localization and resection margins while minimizing collateral trauma. Simulation-based training, competency benchmarks, and international registries can consolidate quality and safety. Equitable access across resource-limited settings, family-centered care, and long-term surveillance for functional, psychosocial, and oncologic outcomes remain priorities. Finally, telemedicine-enabled follow-up and data-driven decision support promise precision and continuity of care.

## Introduction and background

Approaches and techniques for pediatric chest diseases have evolved over the past few years [1]. This occurred as a result of the advancement and the better understanding of the technology and a deeper learning of the specific anatomical, physiological, and psychological challenges presented by children [2].

Children are not just "small adults"; they require specialized, individualized care due to their specific and different anatomy, physiology, and immature immune system. For instance, because infants and children have small thoracic spaces, smaller airways, and more cartilaginous and pliable chest walls, thoracic surgical techniques designed for adults frequently need to be significantly changed when applied to children [1,2].

The improvement of anesthetic techniques, robotic-assisted thoracic surgery, and video-assisted thoracoscopic surgery (VATS) has significantly improved postoperative outcomes and decreased morbidity [3,4]. However, these methods have problems, including the need for a high degree of skill, the need to modify equipment for small spaces, and the need to handle particular airway problems [2,5]. Recent research highlights the necessity of multidisciplinary cooperation, continued training, and ongoing development and evaluation of novel techniques tailored especially for kids [1]. Continued and evolving research, technological improvement, and international collaboration are vital to optimizing outcomes and shaping the future of care for these advances [5]. This narrative review concentrates on advances in this field, provides a neonatal-focused synthesis (airway anomalies, congenital lung lesions, esophageal and diaphragmatic surgery, peri-insufflation physiology, NICU/ECMO interfaces), and integrates emerging technologies. We also summarize pediatric enhanced recovery after surgery (ERAS)/perioperative practices, long-term outcomes, and implementation/equity issues that shape real-world adoption.

## Review

Methodology

This narrative review aims to synthesize practice and future directions in pediatric thoracic surgery. We performed targeted literature searches in PubMed/MEDLINE, Scopus, Web of Science, and the Cochrane Library for articles published January 2015-January 2025.

Search strings combined MeSH terms and free-text keywords related to pediatric thoracic surgery (e.g., “pediatric/paediatric thoracic surgery,” “minimally invasive/VATS/robotic,” “airway disorders,” “congenital lung malformations,” “chest wall reconstruction,” “thoracic oncology,” “pediatric lung transplantation”), using Boolean operators to iteratively refine retrieval.

Eligibility and selection were pragmatic and relevance-based, consistent with a narrative synthesis. We included English-language publications addressing patients 0-18 years and thoracic surgical care, spanning clinical studies (randomized and observational), case series, high-quality reviews, consensus statements, and guidelines. We excluded adult-only reports, conference abstracts without full text, and items lacking substantive clinical or methodological content. Case reports and expert opinions were considered when illustrative of emerging techniques or controversies.

Synthesis was thematic and non-quantitative. Evidence was organized across domains: airway disorders and surgical management; congenital thoracic anomalies (with emphasis on lung malformations); chest wall reconstruction; pediatric lung transplantation; thoracic oncology; and perioperative care/outcomes (including complications and follow-up), as well as emerging technologies and research priorities. No meta-analysis was undertaken.

Recognizing the breadth and heterogeneity of the literature, we did not apply PRISMA or formal risk-of-bias tools. Instead, during synthesis, we weighted evidence by study design rigor, sample size, follow-up adequacy, outcome validity, and plausibility, giving proportionally greater emphasis to systematic reviews, meta-analyses, and well-designed prospective studies while transparently integrating relevant observational data and expert consensus.

Current landscape and advances in minimally invasive surgery

Over the past few years, the approaches for pediatric chest conditions have evolved and improved. Classic open thoracic procedures have been replaced by minimally invasive operations. These minimally invasive techniques have been adjusted and used for even the smallest anatomical challenges and structures in infants and children, significantly widening the field and types of thoracic surgeries feasible in the pediatric population [4,6].

VATS is now a well-established approach for different diseases, including empyema, congenital malformations, and mediastinal tumors [4,7]. Studies have highlighted that VATS in children is associated with less observed postoperative pain, shorter hospital stays, better cosmetic outcomes for patients, and lowered long-term morbidity compared to open surgery, which highlights its importance [7,8]. Pogorelić et al. [7] found that early decortication for pediatric empyema using VATS led to better recovery times, and discharge conducted in India showed that the importance of VATS for pediatric empyema, as it is associated with low morbidity and mortality rates, minimal need for redo intervention, and a reduced hospital stay time, consistently aligning with global findings on MIS approaches [2,9].

The creation of customized surgical instruments has also contributed to the growth of minimally invasive surgery. Energy devices, staplers, and tiny endoscopic devices made specifically for children that have increased safety, accuracy, and effectiveness during difficult thoracic procedures and helped in the improvement of all approaches [6].

Robotic-assisted thoracic surgery (RATS) is developing in pediatric care, offering good visualization and outcomes. However, its use is currently limited to specialized centers, and more clinical trials are needed to evaluate its safety, cost-effectiveness. Ongoing challenges include optimal anesthetic approaches, management of complex congenital conditions, and the adaptation of minimally invasive techniques for neonates and infants, which need continued and specialized training [10].

**Figure 1:**
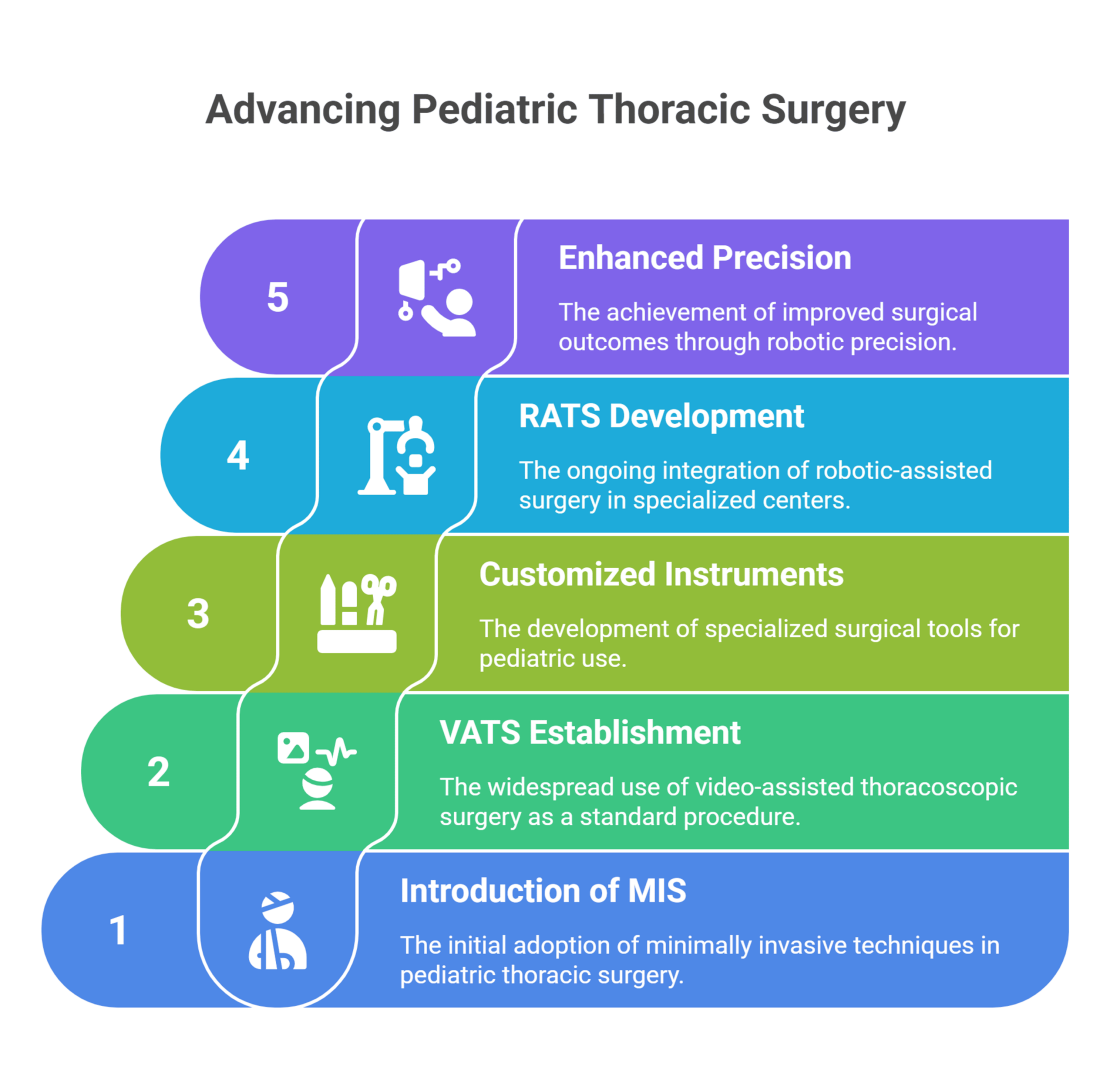
Current landscape of pediatric thoracic surgery. VATS: Video-assisted thoracoscopic surgery; RATS: Robotic-assisted thoracic surgery. MIS: Minimally invasive surgery. Figure Credit: Momen Abdelglil. Source: [2-9].

Recent multi-center clinical studies and institutional reviews indicate that robotic platforms enhance surgical precision, particularly for complex cases such as posterior mediastinal mass excision, esophagectomy for corrosive strictures, and tumor resections. Robotic systems provide improved three-dimensional (3D) visualization and greater instrument dexterity within confined thoracic spaces, resulting in high rates of complete tumor resection and low conversion rates to open surgery (Figure 1) [3,11,12]. 

Advances in neonatal thoracic surgery

The field of neonatal thoracic surgery now encompasses both traditional open techniques and minimally invasive approaches, offering tailored interventions for rare, life-threatening congenital conditions such as congenital cystic lung malformations, congenital diaphragmatic hernia, esophageal atresia, and complex cardiac anomalies [4].

The unique physiology of newborns including immature lung development, limited cardiopulmonary reserve, and delicate anatomy necessitates a specialized approach [2,13]. Neonatal thoracic procedures encompass a broad spectrum of conditions, including congenital diaphragmatic hernia, esophageal atresia with tracheoesophageal fistula (EA/TEF), congenital cystic adenomatoid malformations, and various cardiac anomalies [14,15].

The advances toward minimally invasive surgical techniques have transformed neonatal thoracic surgery, with thoracoscopy becoming increasingly prevalent despite. Thoracoscopic approaches introduce many advantages, including reduced tissue trauma, decreased postoperative pain, shorter hospital stays, and improved cosmetic outcomes [2,13]. However, these interventions have many challenges to be applied globally, including limited working space, technical complexity requiring steep learning curves, and physiological concerns related to single-lung ventilation and CO2 insufflation effects on cardiovascular function [13].

Hospital mortality rates range from 8.1% to 14.3% for neonatal cardiac procedures, as highlighted by many articles, with notable improvements observed over the past decade. Reported neonatal cardiac surgery mortality varies widely because cohorts differ by procedure complexity, case-mix, and institutional resources [15,16]. For specific diseases like esophageal atresia repair, minimally invasive approaches demonstrate comparable outcomes to open intervention, though some studies suggest higher reintervention rates with thoracoscopic techniques [14,17].

**Figure 2:**
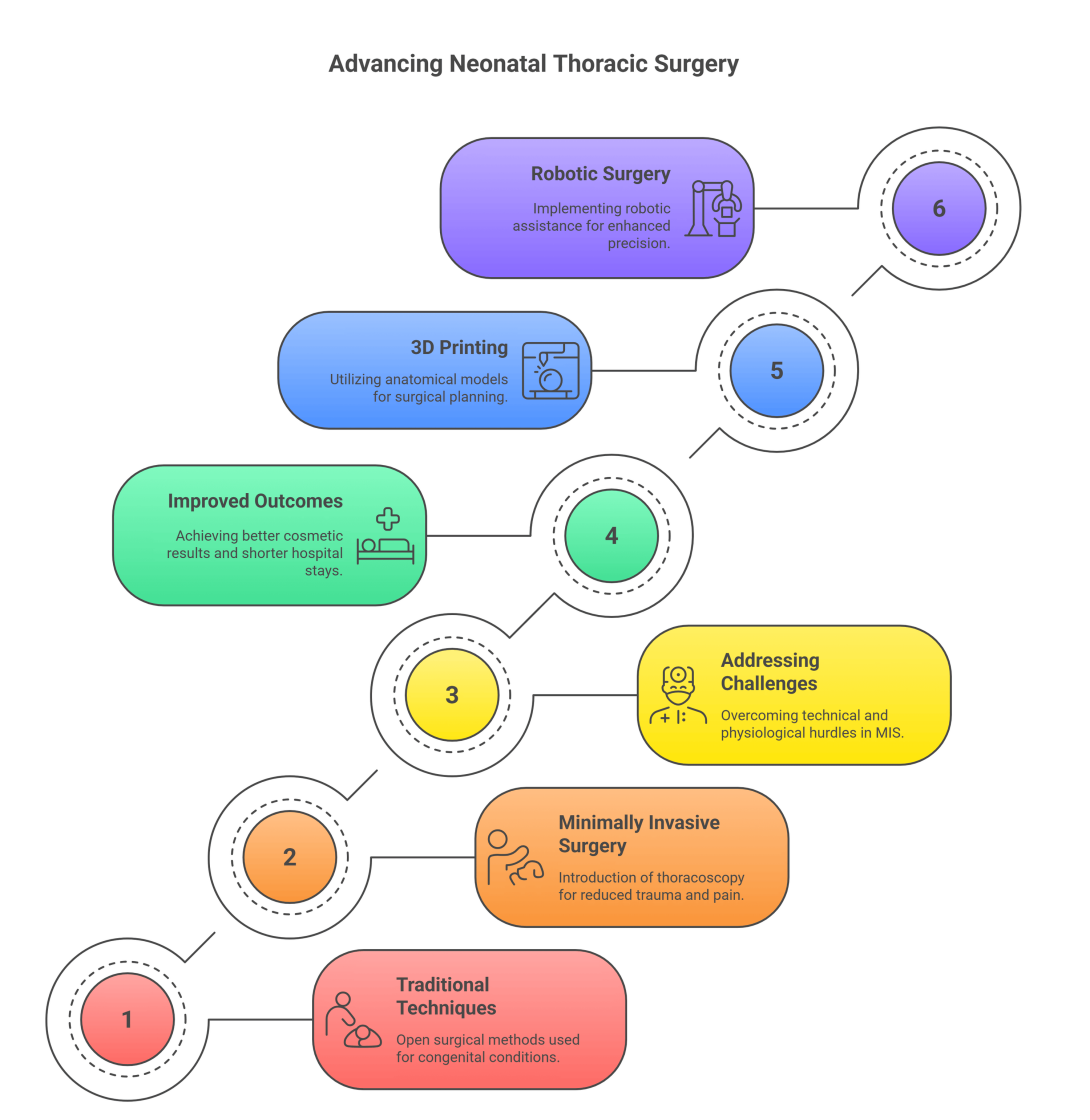
Advances in neonatal thoracic surgery. Figure credit: Momen Abdelglil. Source: [13-17].

3D printing technology helps us in the creation of patient-specific anatomical models for preoperative planning and surgical rehearsal, particularly for complex cases involving airway malformations and cardiac anomalies [18,19]. Robotic-assisted surgery has demonstrated feasibility and potential benefits, including enhanced precision and reduced invasiveness (Figure 2) [20]. 

Treatment of congenital lung malformations

Many changes have been introduced to the treatment of congenital lung malformations (CLMs), transforming the treatment of these conditions in neonates and children. Studies of neonates undergoing thoracoscopic resection for symptomatic CLMs demonstrate good clinical outcomes, including minimal intraoperative blood loss, low complication rates, and no thoracic deformity after surgery. These outcomes indicate that with proper patient selection and surgical expertise, thoracoscopic removal of congenital lesions offers a safe and effective alternative [21].

Another significant advancement is the use of lung-sparing operations and techniques, such as thoracoscopic segmentectomy. VATS segmentectomy preserves more lung tissue while effectively removing the disease or malformation. Recent research has shown that in a cohort of children with CLMs, thoracoscopic segmentectomy had outcomes comparable to those of the thoracoscopic type, with a very low conversion rate to open surgery [22].

Comparative research between thoracoscopic and open procedures for CLM management in children has highlighted the advantages of the minimally invasive approaches. Propensity-matched multicenter analyses showed that thoracoscopic lobectomy and open lobectomy have similar 30-day clinical outcomes, but thoracoscopic surgery is associated with a significantly shorter hospital length of stay. Over the last few years, the proportion of thoracoscopic cases has significantly increased, reflecting growing surgeon confidence and the accumulating evidence of safety and efficacy for thoracoscopic approaches in pediatric lung surgery [23].

More recently, innovative surgical techniques, such as HuaXi thoracoscopic anatomical lesion resection (HX-TALR), have been developed. Large retrospective studies showed that HX-TALR is safe, feasible, and free of many complications, though it has a difficult learning curve and requires specialized training [24].

Thoracic oncology in children

Thoracic oncology in children includes a different and complex group of neoplasms arising within the chest, including the lungs, mediastinum, chest wall, and pleura. Unlike in adults, primary pulmonary tumors are rare in children; most thoracic malignancies are metastatic or stem from non-epithelial tissues. Among neoplastic entities, lymphoma, germ cell tumors, and chest wall sarcomas are common. The different anatomy and broad pathological spectrum of pediatric thoracic tumors necessitate a multidisciplinary and adjusted approach to diagnosis, staging, and management [25,26].

A crucial concern in pediatric thoracic oncology is the potential for long-term functional impairment and skeletal deformity, especially in children requiring extensive chest wall resection or thoracotomy. Studies with long-term follow-up reveal that children undergoing thoracotomy with rib resections face a higher risk of developing scoliosis and restrictive lung disease, leading to measurable impacts on pulmonary function and quality of life. Thoracoscopic and less invasive surgical approaches have demonstrated fewer complications, especially regarding lung function [25].

A multidisciplinary team, including oncologists, radiologists, thoracic surgeons, and rehabilitation specialists, is needed not only for initial intervention but also for ongoing surveillance. Regular follow-up utilizing pulmonary function tests and quality of life assessments identifies children who may benefit from early intervention for skeletal or functional complications. Advances in imaging and minimally invasive surgery continue to refine patient selection and risk assessment, thereby reducing complications [25,27].

Chest wall deformities and reconstruction

Chest wall abnormalities in children, such as pectus excavatum and pectus carinatum, represent the most common congenital diseases affecting the thoracic bone cage. These deformities can lead to significant physiological results including respiratory and cardiovascular impairment as well as psychosocial issues related to body shape and self-esteem. Early identification and multidisciplinary team assessment are essential for developing individualized management strategies [28].

Surgical repair of chest wall deformities in the pediatric population has changed and improved markedly over recent decades. The introduction of minimally invasive techniques, such as the Nuss procedure, has transformed the management of pectus excavatum by enabling sternal elevation via retrosternal bar placement with notably less morbidity than traditional open approaches. Advancements in bar stabilization and sternal elevation strategies, alongside adjunctive thoracoscopy, have improved safety and outcome so patients experience shorter hospital stays, reduced postoperative pain, and excellent cosmetic and functional outcomes [29].

In managing extensive chest wall defects from tumors, trauma, or congenital conditions, reconstruction is adjusted to defect characteristics and patient shape. Techniques include bioprosthetic patches, autologous flaps, synthetic meshes, and implants like titanium plates. While rigid materials offer stability, they risk long-term issues such as stiffness or scoliosis if growth is not highlighted. Hybrid methods using semi-rigid meshes with muscle and skin flaps aim to reduce these risks while preserving chest wall mobility and ensuring good reconstruction [30].

Furthermore, 3D printing technology has started to play a role in preoperative planning and the development of a patient-specific type of implant. While widespread adoption is difficult in many regions, such approaches offer good possibilities for anatomically precise repair, especially in cases with complex deformities. Early clinical experience suggests that 3D-printed prostheses may improve structural support and cosmetic results [31].

Patient outcomes following pediatric chest wall repair are generally good when procedures are performed in specialized centers with multidisciplinary expertise. Large cohort studies and systematic reviews report high patient satisfaction, resolution of functional impairments, and durable correction of these anomalies. Ongoing research into new biomaterials, minimally invasive and robotic-assisted techniques, and individualized surgical timing continues to refine the balance between optimal thoracic mechanics, growth, and cosmetic outcomes in this different patient population [32].

Management of airway disorders 

Recent advancements in difficult airway management include videolaryngoscopy, passive oxygenation, and the use of supraglottic airway devices with flexible bronchoscopes. These techniques aim to reduce intubation attempts or failure in pediatric patients, improving success rates even in complex cases [33].

Surgical intervention for airway diseases is highly individualized, involving operations such as aortopexy for tracheomalacia and tracheal resection for localized disease. The success of these surgeries in children largely depends on precise technique and intraoperative visualization, with most experienced centers reporting success rates and low complication incidences [34].

Management of TEFs has evolved with the application of interventional techniques. Endoscopic placement of stents helps seal the fistula, prevents leakage, and reconstructs the airway, especially useful in challenging cases. Double stenting is indicated for larger or more complex fistulas to optimize therapeutic outcomes [35].

Pediatric lung transplantation

Pediatric lung transplantation may be an intervention for children with end-stage pulmonary diseases such as cystic fibrosis. Recent trends indicate a growing demand for transplants in cases of pulmonary vascular diseases and interstitial lung disease [36].

New surgical approaches, such as transplantation of downsized adult lung segments or lobes, have advanced the field. These techniques accommodate smaller chest space and have become essential in surgical practice, making up to 40% of some centers’ cases. Pulmonary bipartitioning and lobar transplantation now allow the efficient use of donor organs and successful outcomes for smaller recipients [37].

Outcomes in pediatric lung transplantation are generally improving. The worldwide median survival is 5.7 years, but in certain populations, median survival exceeds 10 years with optimal donor selection, perioperative planning, and advanced surgical techniques. Age, underlying disease, and transplant center expertise are major determinants of long-term success [37,38].

Quality of life following pediatric lung transplantation is often substantially improved. Most recipients experience few, if any, limitations by three years after transplant and achieve good health-related quality of life metrics, though ongoing surveillance for graft function and potential complications remains essential to maximize long-term benefits [39].

Postoperative care, complications, and long-term outcomes

Optimal postoperative care in pediatric thoracic surgery involves a multidisciplinary team and the adaptation of enhanced recovery protocols. Important elements include early mobilization, effective pain control, and adequate nutrition to reduce complications and shorten hospital stays. Pain management is also highlighted due to the risk of respiratory issues, with recent studies supporting a multimodal analgesia approach to improve pain relief, reduce side effects, and support faster recovery [40].

Complications following pediatric thoracic procedures remain an important concern for doctors and patients. Common complications include chest infections, heart failure, wound infections, and arrhythmias. The incidence and severity of these problems are influenced by factors such as the complexity of the procedure, the duration of cardiopulmonary bypass when used, and patient-related conditions [41].

Future directions and research priorities

RATS has emerged as a promising approach that can contribute to the future of surgical intervention, with recent studies demonstrating significant potential for complex pediatric cases [3].

The future directions in pediatric and neonatal thoracic surgery contain several areas of development. Enhanced perioperative care and assessment systems incorporating real-time physiological feedback and predictive analytics may improve patient selection and surgical planning [42,43]. 

The use the adoption of artificial intelligence and machine learning in surgical practice and training represents another critical research priority, with predictive models showing remarkable accuracy in forecasting surgical outcomes and problems [44]. ERAS protocols, classically applied in adult patients, are gaining momentum in pediatric thoracic surgery, with early evidence suggesting reduced length of stay and improved postoperative recovery [45]. 

Simulation-based training, together with virtual and augmented reality, is reshaping pediatric thoracic surgical education. Recent advances in immersive VR/AR platforms enhance technical performance, enable realistic preoperative rehearsal, and measurably shorten the learning curve for complex procedures [46]. 3D printing technology is enabling the creation of patient-specific anatomical models [47]. 

Personalized medicine approaches are emerging as a crucial research priority in pediatric thoracic surgery, particularly in the management of congenital anomalies and thoracic malignancies. Genetic and molecular profiling technologies are enabling tailored treatment approaches that address individual patient characteristics and disease-specific factors. The development of bioengineered materials and 3D bioprinting for chest wall reconstruction represents a significant advancement in personalized surgical solutions [48,49]. 

**Figure 3:**
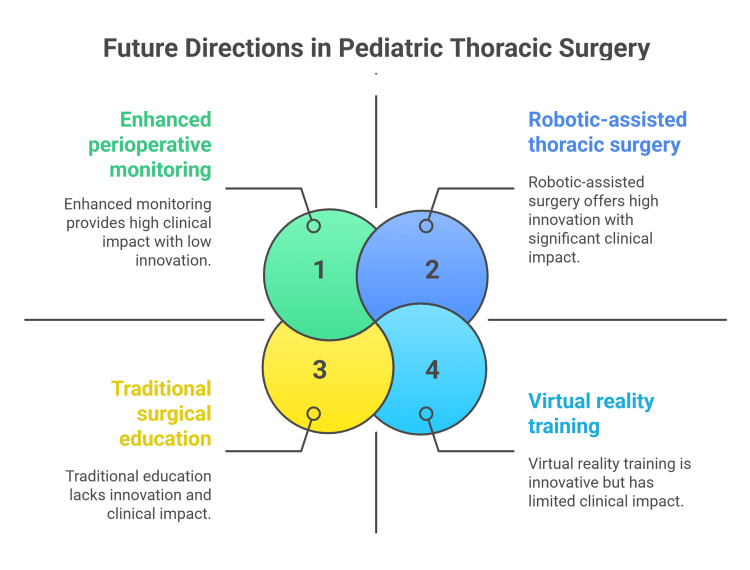
Future directions for pediatric thoracic surgery. Figure credit: Momen Abdelglil. Source: [47-50]

Telemedicine and digital health technologies are expanding access to specialized pediatric thoracic surgical care, particularly in underserved regions and resource-limited settings. Recent studies demonstrate high diagnostic accuracy and patient satisfaction with telemedicine consultations for pediatric surgical conditions [50]. The integration of artificial intelligence into telemedicine platforms is enhancing diagnostic capabilities and enabling remote surgical guidance (Figure 3) [51]. 

## Conclusions

Minimally invasive and robotic techniques, improved imaging, and pediatric-specific perioperative care are reshaping pediatric and neonatal thoracic surgery. Extending VATS and RATS to neonates shortens recovery, reduces pain, improves cosmesis, and preserves outcomes through multidisciplinary planning, instrumentation, and pathways.

Emerging technologies such as 3D printing, simulation, telemedicine, and data-driven decision support promise personalized, equitable care while strengthening training and quality oversight. Key priorities are expanding access, adapting adult-derived protocols to children, and advancing ERAS, airway, oncology, and transplantation outcomes.
